# A Study of Ta_2_O_5_ Nanopillars with Ni Tips Prepared by Porous Anodic Alumina Through-Mask Anodization

**DOI:** 10.3390/nano12081344

**Published:** 2022-04-14

**Authors:** Alla I. Vorobjova, Daria I. Tishkevich, Elena A. Outkina, Dmitry L. Shimanovich, Ihar U. Razanau, Tatiana I. Zubar, Anastasia A. Bondaruk, Ekaterina K. Zheleznova, Mengge Dong, Dalal A. Aloraini, M. I. Sayyed, Aljawhara H. Almuqrin, Maxim V. Silibin, Sergey V. Trukhanov, Alex V. Trukhanov

**Affiliations:** 1 Micro- and Nanoelectronics Department, Belarusian State University of Informatics and Radioelectronics, 220013 Minsk, Belarus; vorobjova@bsuir.by (A.I.V.); outkina@bsuir.by (E.A.O.); shdl@tut.by (D.L.S.); katenickerd@gmail.com (E.K.Z.); 2 Scientific-Practical Materials Research Centre of NAS of Belarus, 220072 Minsk, Belarus; ir23.by@gmail.com (I.U.R.); fix.tatyana@gmail.com (T.I.Z.); bondaruk625@gmail.com (A.A.B.); sv_truhanov@mail.ru (S.V.T.); truhanov86@mail.ru (A.V.T.); 3 Laboratory of Single Crystal Growth, South Ural State University, 454080 Chelyabinsk, Russia; 4 Department of Resource and Environment, Northeastern University, Shenyang 110819, China; mg_dong@163.com; 5 Department of Physics, College of Science, Princess Nourah Bint Abdulrahman University, P.O. Box 84428, Riyadh 11671, Saudi Arabia; daalorainy@pnu.edu.sa (D.A.A.); ahalmoqren@pnu.edu.sa (A.H.A.); 6 Department of Physics, Faculty of Science, Isra University, Amman 1162, Jordan; dr.mabualssayed@gmail.com; 7 Department of Nuclear Medicine Research, Institute for Research and Medical Consultations (IRMC), Imam Abdurrahman bin Faisal University (IAU), Dammam 31441, Saudi Arabia; 8 Scientific and Technological Park of Biomedecine, I.M. Sechenov First Moscow State Medical University, 119991 Moscow, Russia; sil_m@mail.ru

**Keywords:** nanopillars, nanotips, porous anodic alumina, nanoparticles, superparamagnetic behavior

## Abstract

The paper discusses the formation of Ta_2_O_5_ pillars with Ni tips during thin porous anodic alumina through-mask anodization on Si/SiO_2_ substrates. The tantalum nanopillars were formed through porous masks in electrolytes of phosphoric and oxalic acid. The Ni tips on the Ta_2_O_5_ pillars were formed via vacuum evaporation through the porous mask. The morphology, structure, and magnetic properties at 4.2 and 300 K of the Ta_2_O_5_ nanopillars with Ni tips have been studied using scanning electron microscopy, X-ray diffraction, and vibrating sample magnetometry. The main mechanism of the formation of the Ta_2_O_5_ pillars during through-mask anodization was revealed. The superparamagnetic behavior of the magnetic hysteresis loop of the Ta_2_O_5_ nanopillars with Ni tips was observed. Such nanostructures can be used to develop novel functional nanomaterials for magnetic, electronic, biomedical, and optical nano-scale devices.

## 1. Introduction

Magnetic nanoparticles (MNPs) of Fe, Ni, and Co, as well as their chemical compounds have the potential to create high-density magnetic memory, single-electron devices, biosensors, nanoelectrodes, and other devices [1,2,3,4]. Many research groups are actively developing magnetic nanocomposites on the basis of nickel, cobalt, iron, iron oxide, and ferrite nanoparticles for biomedical applications [5,6,7]. The MNPs can be controlled inside an organism by using external magnetic field strength gradients. The possibility of such control makes it possible to use them for various clinical tasks, including magnetic resonance imaging (MRI), targeted drug delivery, and magnetic thermotherapy [8,9].

Our analysis has shown that most research works discuss “free” nanoparticles in the form of powders, suspensions, and aerosols. However, the MNPs usually constitute a part of a film (2D systems) or bulk material (3D systems) in real applications. Simple compaction of the MNPs often leads to a loss or a substantial change in their unique physical characteristics and functional features, even when the particles have a protective coating. Therefore, the optimal structure of an MNPs-containing material can be thought of as single-domain MNPs of a narrow size distribution, which were homogeneously dispersed in a nonmagnetic dielectric matrix. For example, in work [10], maghemite nanoparticles-decorated anodic alumina nanotubes were used for the fluorescent detection of cathepsin B. The diameter of the nanotubes was about 80 nm, the length was about 1 μm, and the diameter of the magnetic nanoparticles was 10 nm. This is a nanocomposite made of 2D/3D material as well.

The chemical and physical properties of the MNPs strongly depend on the synthesis approaches and chemical structures. The formation of nanostructure arrays of various shapes on the base of the MNPs is a promising novel approach of the problem for other applications, such as temperature sensors, thermal energy storage devices, and energy harvesters.

Research in the field of nanostructure array formation is mostly concentrated on carbon nanotubes (CNT) [11,12] and magnetic or semiconducting nanowires or nanodots [13,14,15]. However, the physical vapor deposition (PVD) [11] and chemical vapor deposition (CVD) [16] methods of their synthesis require high temperatures. In other methods, high-temperature annealing is used [17].

As a result, low-temperature synthesis methods are constantly attracting researchers’ attention. The chemical methods for the synthesis of the MPNs from metal-containing compounds (MCC) are the most widely used ones [10,18,19]. Ultrasonic MCCs decomposition, MCCs reduction by various reducing agents, radiation–chemical metal ion reduction, sol–gel technique, MNPs synthesis on a water–air phase interface (Langmuir–Blodgett technology) [20,21], and some other less well-known and investigated methods [22] are included.

One of the popular modern approaches is based on the use of a porous template [23,24,25]. Such templates are completely or partially removed after the nanostructure synthesis. Polycarbonate or thin, porous anodic alumina (TPAA) membranes are usually used for this purpose [26,27,28]. TPAA is almost an ideal template because of its unique structure: it is an ordered matrix of cylindrical pores perpendicular to the surface. The possibility of controlling the structure characteristics by regulating the anodizing parameters such as voltage and duration as well as the electrolyte type and composition [29,30,31] is another advantage of such templates. Applying porous alumina as a template allows forming arrays of vertically ordered NPs with uniform geometrical parameters (diameter, length, and density), which can be readily controlled in a wide range of sizes [32,33]. Additionally, porous alumina is the heat-resistant material that allows carrying out various experiments at high temperatures [34,35]. Furthermore, the TPAA synthesis is simple and inexpensive. It enables the use of a variety of functional substrates, including silicon, sitall, glass, or bulk aluminum [36,37]. 

MNPs can be synthesized using microelectronics methods on the surface of nanopillars and on the surface or inside of nanotubes. As a result of the interactions in heterogeneous solid-state composites, new properties and possibilities can arise in such systems. For example, the authors of [38,39] reported that tantalum oxide nanopillars systematically self-organized in alumina pores can be formed under certain conditions during TPAA formation in two-layer systems consisting of a relatively thin film of tantalum and a relatively thick film of aluminum.

Within certain limits, the magnetic properties of materials on the base of MNPs can be controlled by changing their size, shape, composition, and structure. However, it is often not feasible to control these parameters during the synthesis of nanoparticles of similar size and chemical composition. As a result, the properties of the same type of nanomaterial can differ significantly. Hence, only complex studies of both the formation peculiarities (mechanisms) and morphological, structural, electronic, and magnetic properties of MNPs with a narrow size distribution allow for formulating necessary synthesis recommendations and finding new promising application directions for the nanomaterials.

For biomedical applications, composite magnetic structures on the basis of MNPs should have three important properties: (1) they should be small (from units to several tens of nm) with a narrow dispersion of topological parameters; (2) they should not assemble into bundles or clusters because magnetic particle agglomeration is undesirable in biomedicine; and (3) they should be controllable by a magnetic field during medical treatment or diagnostics, as for example, during magnetic resonance therapy [11]. After that, the MNPs should be passive as the time of their removal from the human organism can be up to several days. In such cases, superparamagnetic particles of iron oxide coated with gold or platinum are usually used [40]. There are various methods of such superparamagnetic particle synthesis [41], mostly chemical ones. Core–shell particle materials and their synthesis are costly and laborious.

Structured media of this type were traditionally formed using such nano-scale processes as magnetic material etching through a mask, explosive lithography, and vacuum deposition of a magnetic material through a shadow mask [42]. In the work [43] are proposed two sequences for the formation of a metal/porous alumina composite obtained via vacuum deposition. The first sequence is a continuous metal deposition process, and the second is an interrupted deposition scheme, which consists of metal deposition and the mechanical removal of metal from the surface using adhesive tape. In [44], structures of two types were obtained: Ni nanohoneycomb chain structures and Ni nanostructures in the form of double rings. The structures were formed via metal sputtering onto the surface of TPAA substrates. A deposition method with a change in the angle of inclination was used for accurate deposition of a nanothin Ni layer (no more than 10 nm) on a porous alumina. The deposition method using an unusual shadow mask made from porous alumina for the magnetic matrices fabrication with magnetic inclusions of about 60 nm on an area of 1 cm^2^ is proposed in [45]. In this study, a 300 nm thick alumina membrane with a pore diameter of 60 nm and a pore density of 10^10^ cm^2^ was used. Using this method, a Fe layer of 15 nm thick was deposited on a MgO substrate by electron beam evaporation through pores. Then, the membrane was removed in a 10% NaOH solution. In the alumina mask, the pores had a fairly uniform size, with an average diameter of 61 ± 6 nm. The size of Fe nanodots, along with a narrow distribution range, was 58 ± 8 nm. Thus, the combination of a thin mask and directional flow minimizes the shadowing effect and can provide structural uniformity. The authors believe that such methods of physical deposition, despite the complexity of controlling the deposition process, are quite simple to form homogeneous nanostructures. However, there is one significant problem, such as the complex fabrication and difficult work (manipulation) with a thin mask (300 nm) of alumina with through pores.

The advantages of the presented methods are that it is possible to use industrial semiconductor production equipment, which allows the processing of various materials on standard substrates with high reproducibility. The disadvantages are the use of expensive processes for creating high-precision masks for metal deposition. In addition, with a decrease in the size of the elements, the uniformity of deposition over the area of the substrate deteriorates, and to fill nanosized pores, it is necessary to refine the processes of metal-inclined deposition, which complicates the control of deposition processes. 

Developing the TPAA pore-filling method, we propose a different anodic dielectric as the filling material: tantalum oxide with further deposition of magnetic nickel on it. In such a case, the initial structure is a two-layer composition including Al as the top layer and Ta as the bottom layer. The method proposed allows the obtaining of ordered pillar “oxide-magnetic” structures that have been actively studied recently because of a wide range of possible applications in nanoelectronics and biomedicine [46,47,48,49]. We propose a method that is closer to industrial production conditions and compatible with silicon micro- and nanoelectronic device production technology. 

## 2. Experimental

The experimental samples were prepared in five stages, as described below. The experimental samples were double-layer thin-film structures of aluminum and tantalum deposited on standard silicon (Si/SiO_2_) substrates. 76 mm in diameter. 

In the first step, polycrystalline films of Al and Ta with the grains close in size to their thickness were deposited by electron-beam evaporation separately or in a single vacuum cycle using a 01NE-7-004 setup “Oratorio-9” (Kaliningrad machine-building factory, Kaliningrad, Russia). The Al layer served as a porous aluminum oxide (Al_2_O_3_) formation. The Ta layer was used for tantalum oxide (Ta_2_O_5_) pillar formation. It also served as an adhesion sublayer and the bottom electrode. The thickness of the Ta film was controlled by a witness. The thickness of the Al layer was controlled using a quartz microbalance sensor. The parameters of the vacuum deposition of the Al and Ta thin films are listed in Table 1.

In the second step, thin films of porous anodic alumina Al_2_O_3_ (TPAA) with an ordered structure were prepared by a two-step anodization method in a combined regime. First, a potential sweep at a constant rate up to a set value was used. Then, the potential was kept constant. The samples of type I were prepared using a 4% aqueous solution of oxalic acid (C_2_H_2_O_4_) under a stationary oxide formation voltage of 40 V and an electrolyte temperature of 14 °C. The samples of type II were prepared using a 4% aqueous solution of phosphoric acid (H_3_PO_4_) under a stationary oxide formation voltage of 80 V and an electrolyte temperature of 14 °C. The anodization was carried out in a thermostatic two-electrode cell with magnetic stirring using a graphite cathode.

In the third step, after the through anodization of the Al film and reaching the Ta film, anodic oxidation was continued in the same electrolyte under the voltage sweep conditions with a rate of 0.1 V/s up to 70 V for the oxalic electrolyte and 90 V for the phosphoric electrolyte.

We used a P-5827 potentiostat–galvanostat (Measuring Instruments Factory, Gomel, Belarus) to control the parameters of the anodization processes. A specially developed setup was used to synchronously record the time kinetics of the main anodizing parameters, including current and voltage, and the chronovoltamperometric (CVA) diagrams during the anodization.

Table 2 describes the parameters of the samples obtained after the third stage and the procedures of their preparation (initial matrices—TPAA).

**Figure 1:**
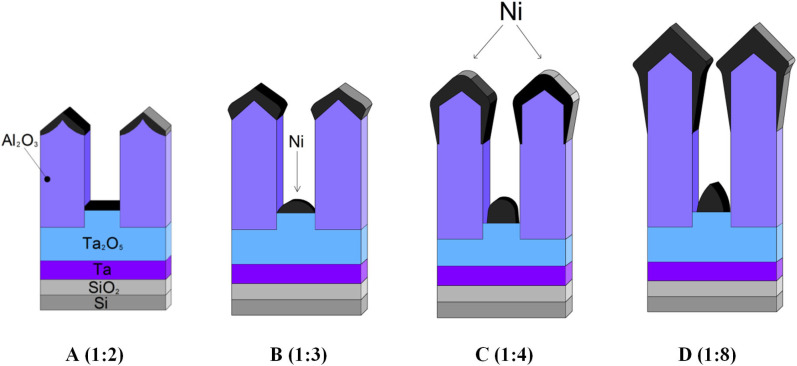
Schematic representation of possible shadowing effects of the Ni coating deposited onto a thin porous anodic film. The thickness of the Ni coating increases and the ratio of the pore diameter to the oxide thickness decreases from (**A**–**D**) [50].

In the fourth stage, a nickel film with a thickness of 90 ± 10 nm was deposited on the surface of the samples. The thickness was determined based on the previous experimental data and theoretical recommendations provided in [50] (Figure 1). The thickness of the coating is chosen based on the TPAA parameters, namely, pore diameter and oxide thickness. When the ratio of the pore diameter to the oxide thickness is ≤1:8, the thickness of the metal deposited should be less than 2*d*, where *d* is the Al_2_O_3_ pore diameter.

Nickel films were deposited in a magnetron deposition system on the base of a modernized vacuum setup, “Oratorio-5” (Kaliningrad machine-building factory, Kaliningrad, Russia). A target made of Ni with a purity of 99.95% was used for the deposition. The deposition regime was experimentally adjusted to obtain a low controllable deposition rate. The target voltage was 400 V. The temperature of substrate varied between 280 and 300 °C. The residual gas pressure in the chamber was 13.3 Pa. The deposition rate was about 30 nm∙min^−1^ under such conditions.

In the fifth stage, alumina selective chemical etching was carried out in the electrolyte containing H_3_PO_4_ (6 mass%) and CrO_3_ (1.8 mass%) at a temperature of 85 °C for 3 min. As a result, nickel remained only on top of the Ta_2_O_5_ pillars in the form of small tips.

The morphology of the Ta_2_O_5_ pillars with Ni tips was studied by scanning electron microscopy (Philips XL 30 S FEG–Ku Leuven, Leuven, Belgium) at a 20 kV accelerating voltage in the SE regime. A statistical analysis of the Ta_2_O_5_ pillars was performed using a simple method described in detail in [51]. Smart SEM software was applied to at least three SEM images to calculate the size distribution. X-ray diffraction (XRD) investigation of the prepared samples was carried out using a diffractometer (DRON-3M–NPO Burevestnik, St. Petersburg, Russia) with Cu-Kα radiation at 300 K temperature (λ = 1.542 Å). 

The magnetic parameters were measured in the temperature range of 4.2–300 K by a Liquid Helium Free High Field Measurement System (VSM–Cryogenic Limited, London, UK) [52]. The applied magnetic field was ±2 T; the specific magnetization measurement precision *σ* was ±0.01 A·m^2^·kg^−1^.

## 3. Results and Discussion

### 3.1. Mechanism of the Ta_2_O_5_ Pillar Growth

**Figure 2:**
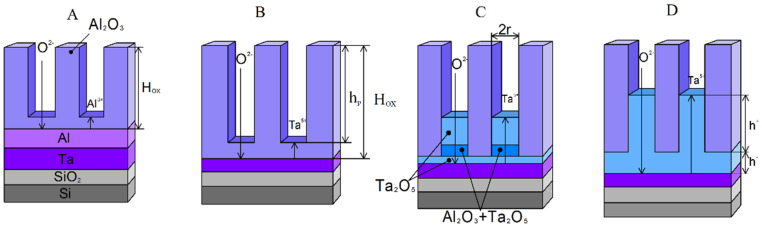
Schematic diagram of the main steps for nanopillars forming from Al/Ta metal sputter-deposited layers on a Si substrate: (**A**) porous anodizing of the Al film to a certain depth; (**B**) porous anodization of the Al film to Ta film; (**C**) anodization of a Ta layer at the Al anodizing voltage (U_Al_); (**D**) formation of Ta_2_O_5_ pillars at Ta anodizing voltage (U_Ta_).

The scheme in Figure 2 explains the stages of nanopillar formation during the anodization of a two-layer thin-film Al-Ta composition in a combined regime. First, the anodization is carried out under a potential sweep at a constant rate. After reaching the potential value set, the anodization is continued under potentiostatic conditions. During stage A, the aluminum film is porously anodized to a certain depth. In stage B, the porous anodization of aluminum reaches the Ta film. Stage C is the Ta film anodization under the voltage of aluminum oxide formation (U_Al_). In stage D, Ta_2_O_5_ pillars are formed under the voltage of Ta anodization (U_Ta_); reanodization takes place.

The size of the Al_2_O_3_ cells and, hence, the distance between the pillars are proportional to the aluminum formation (anodization) voltage. Amorphous Ta oxide fills the central part of the cells with porous Al_2_O_3_ during the reanodization (stages C and D). Matrix pore walls consist of amorphous alumina. The depth of the pore filling with Ta_2_O_5_ is proportional to the formation voltage during the porous anodization of the aluminum layer (stage C). It also depends on the final stage of the aluminum through anodization. The Ta_2_O_5_ pillars formed in stage D repeat the shape of the natural Al_2_O_3_ porous mask (TPAA). The height of the pillars is proportional to the tantalum reanodization voltage (stage D).

**Figure 3:**
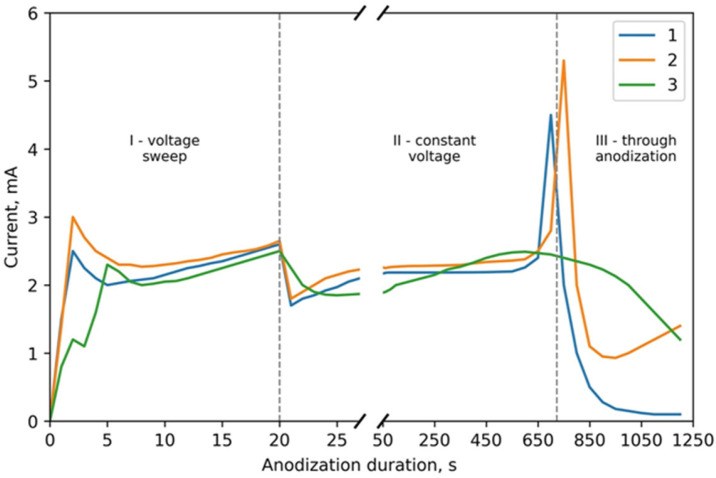
CVA curves of the simultaneous anodic treatment of the two-layer thin-film composition of Al (1000 nm) on Ta (400 nm) for type I samples: **1**—sample No. 1; **2**—sample No. 2; **3**—Al only (1000 nm).

Figure 3 depicts the CVA curves of the simultaneous anodic treatment of the two-layer thin-film composition of Al (1000 nm) on Ta (400 nm) in the 4% aqueous oxalic acid solution. Curve 1 corresponds to sample 1 of type I. Curve 2 shows the anodization of sample 2 of type I. Curve 3 is a comparison curve depicting the usual porous anodization of Al of the same thickness under the same conditions. Anodization was performed in a combined regime. Curves 1 and 3 correspond to the anodization under a voltage sweep from 0 to 40 V at a rate of 2 V·s^−1^ and then under a constant voltage of 40 V. Curve 2 was obtained under the conditions of the potential sweep from 0 to 40 V at a rate of 2 V·s^−1^ and then up to 70 V at a 0.1 V·s^−1^ rate.

**Figure 4:**
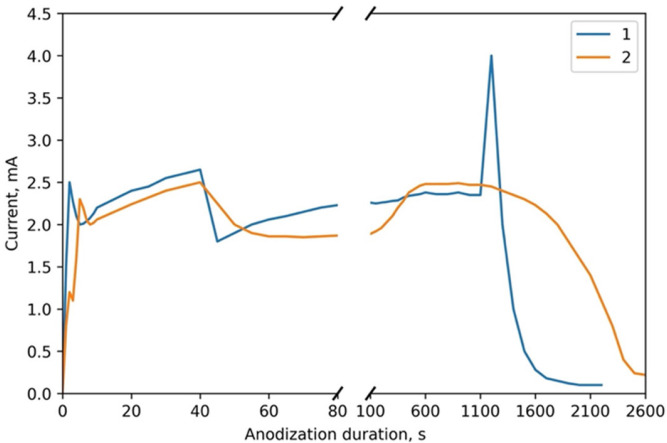
CVA curves of the simultaneous anodic treatment of the two-layer thin-film composition of Al (100 nm) on Ta (400 nm) for type II samples: **1**—sample No. 3; **2**—Al only (1000 nm).

Figure 4 depicts the CVA curves of the simultaneous anodic treatment of the two-layer thin-film composition of Al (1000 nm) on Ta (400 nm) in the 4% phosphoric acid solution. Curve 1 corresponds to sample 3 of type II. Curve 2 is a comparison curve of the usual porous anodization of Al of the same thickness of 1000 nm under the same conditions. Anodization was performed in a combined regime. Curves 1 and 2 represent anodization under a voltage sweep from 0 to 80 V at a 2 V·s^−1^ rate and then under a constant voltage of 80 V.

Analysis of the CVA curves shows that the process consists of three characteristic stages: voltage sweep in stage I, through porous anodization of aluminum film in stage II under constant voltage, and tantalum anodization through pores in the TPAA in stage III. The transition from one layer to another can be easily seen in the CVA curves with an abrupt surge in current. In the region of the voltage sweep (stage I), the curves almost do not differ, as they correspond to the process of reaching a steady-state regime of usual porous aluminum anodization. In the region of constant voltage (stage II), the Ta sublayer assists in a more homogeneous current distribution in the sample. Only in the region of through anodization of aluminum (stage III), an abrupt current surge is observed in curves 1 and 2 of Figure 3 upon the anodization front reaching the Ta film. For the usual porous anodization of aluminum, this stage is characterized by a smooth current decrease to the value of residual current in the porous aluminum oxide formed.

The current increase upon the transition to the Ta layer may relate to the change in the thickness, morphology, and composition of the combined anodic oxide film (AOF) consisting of aluminum oxide and tantalum dioxide. The activated ion concentration increases because of the decrease in the activation energy with the transition of Ta ions into the barrier Al_2_O_3_ layer. After the transition to the Ta layer, it promotes the ion concentration increase on the metal–TPAA boundary. As a result, the ion current density through this surface increases. The Ta ion activation energy decrease upon the transition into the barrier Al_2_O_3_ layer is caused by the synthesis regime: the aluminum AOF was formed under an electric field strength higher than the value necessary for the formation of Ta_2_O_5_. Thus, the AOF resistivity decreases with the transition to Ta.

It is known that [53,54] the cation order changes if the AOF sublayer resistivity is lower than that of the upper metal AOF. It corresponds to the sublayer appearance of metal oxide on the AOF–electrolyte boundary. In our case, the upper metal AOF is a porous 800 nm-thick Al_2_O_3_ oxide. Hence, the sublayer of metal AOF is formed partially below the Al_2_O_3_ and partially inside the Al_2_O_3_ pores above the cell barrier layer as described in the model in Figure 2. The amount of Ta cations becomes higher than that of Al cations, and the oxide color changes. The light gray, transparent anodic aluminum oxide film recolors from blue to violet in the corresponding Ta oxide tone.

The formation of a dense Ta_2_O_5_ oxide layer during the next stage leads to the AOF resistance increasing and the current decreasing to its minimal value. Tantalum starts anodizing when the oxide pore bottom reaches the Al/Ta boundary. The height of the pillar structures can be increased by rising the potential applied after the TPAA formation. Such a method was used to synthesize various nanostructures on various substrates, including thin films of Ta [55], Nb [56], Ti [57,58], and TiN [59].

### 3.2. Morphological and Microstructural Characterization

**Figure 5:**
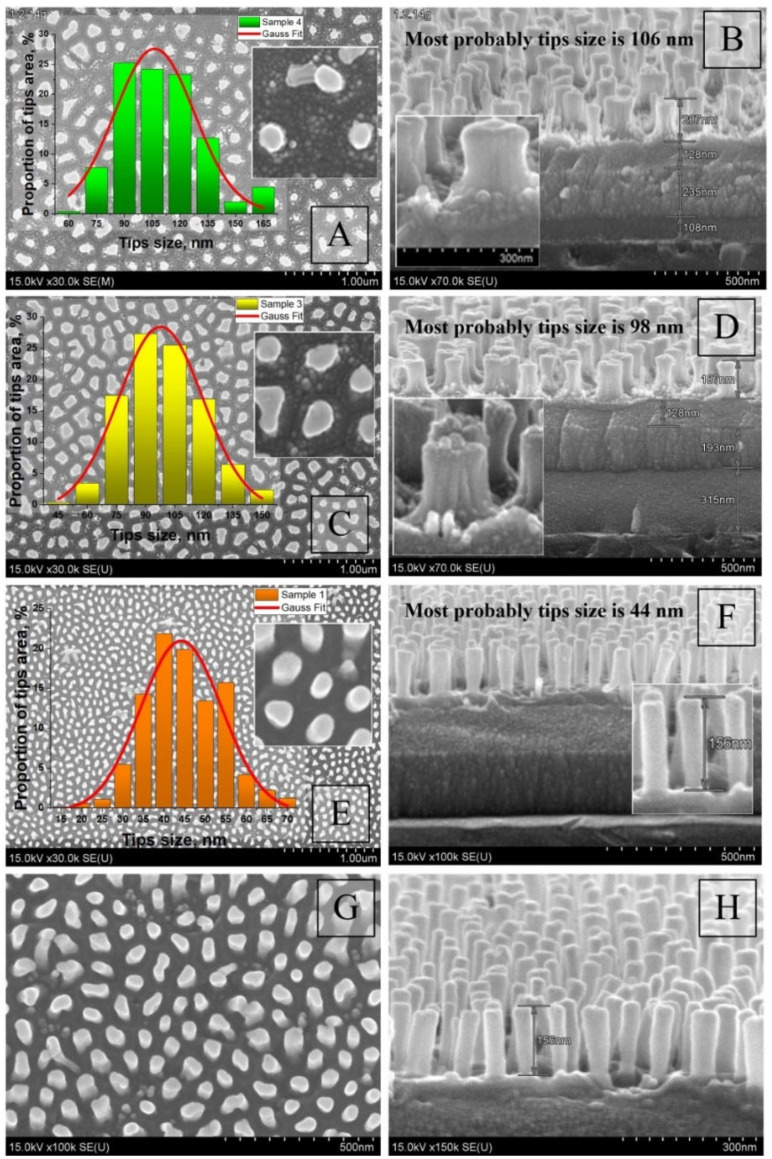
Surface and cross-section SEM images and size histograms for the Ta_2_O_5_ pillars after the Al_2_O_3_ selective etching: (**A**,**B**)—sample 4 of type II; (**C**,**D**)—sample 3 of type II; (**E**–**H**)—sample 1 of type I.

Next, we will present the results of the morphology study for various sample spots obtained at different stages of their synthesis. Figure 5 depicts the surface and cross-section SEM images and size histograms for the Ta_2_O_5_ pillars synthesized under different conditions after the Al_2_O_3_ selective etching.

The pillars have a rather homogeneous shape, with a slight widening both at the bottom and the top parts. The pillars formed under a higher U_Al_ have a larger height and a more developed, loose basis. The images A–D for the type II samples in Figure 5 show that the pillar bottoms were growing through a range of narrow channels in the aluminum oxide pore bottoms, whereas the type I samples shown in images E–H are characterized by nearly ideal cylindrical pillar shapes with smooth sides. Hence, the result primarily depends on the electrolyte type.

**Figure 6:**
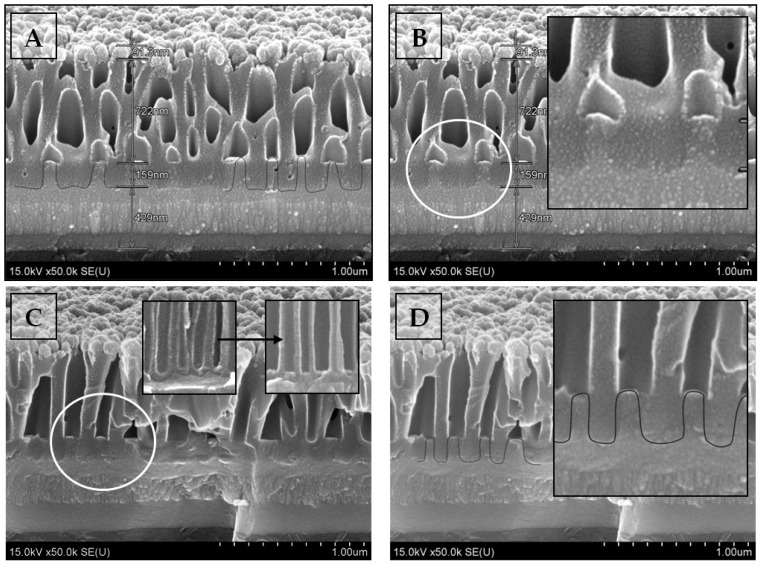
Cross-section SEM images of sample 3 (Si/SiO_2_/Ta/Ta_2_O_5_/TPAA/Ni) after the first stage of double-layer Al/Ta composition anodization and Ni deposition.

Figure 6 shows the SEM images of the samples after the 90 ± 5 nm-thick nickel deposition in the synthesis fourth stage.

Figure 6 depicts the sample cross-section during the initial stage of the Ta_2_O_5_ pillar formation process. Images A and B depict a 30° chip of the sample whose formation was interrupted before the anodization reached the Al/Ta boundary. The images show the cross-section of the Al_2_O_3_ pores with Ta_2_O_5_ pillars growing through the Al_2_O_3_ barrier layer. Images C and D depict a 90° chip of the same sample. The pores have the size typical for Al anodization [60]. The insert in Figure 6B shows an enlarged image of a fragment (white circle) of the cross-section of sample 3 after reaching the Al/Ta interface and Ta_2_O_5_ pillars growing through the Al_2_O_3_ barrier layer (the cross-section was made at an angle of 30°). Inserts in Figure 6C are enlarged images of a fragment of the cross-section of the sample before and after chemical etching of the Al_2_O_3_ barrier layer (cross-section at an angle of 90°). The insert in Figure 6D represents an enlarged image of a fragment (white circle) of the cross-section of the sample after reaching the Al/Ta interface and Ta_2_O_5_ pillars growing through the Al_2_O_3_ barrier layer (cross-section at an angle of 90°).

Figure 6 also shows that Al had been completely anodized, including the residues between the oxide cells. Ta had been anodized to the depth corresponding to the anodization voltage (U_Al_/U_Ta_ = 80/80 V, sample 3, type II). The spherical base of the Al oxide pore cannot be seen anymore. Their bottoms had become flat. The lighter areas below the pores, highlighted by the circles in images B and C in Figure 6, correspond to anodic tantalum oxide that had grown through the aluminum oxide pore bottoms. The size of this area (the height of the Ta pillars h_p_) is 159 ± 5 nm. It is significantly higher than the thickness (h_b_) of the barrier layer for the Al oxide pore bottoms that can be calculated considering the voltage/thickness ratio for the TPAA formation in the aqueous oxalic acid electrolyte as h_b_ = U_Al_ × k_Al_ = 80 V × 1.0 nm/V = 80 nm [61]. We can obtain the Ta anodization constant if we use this ratio for tantalum: k_Ta_ = h_p_/U_Ta_ = 159 nm/80 V ≈ 2.0 nm/V. The value is close to the value obtained in [62]. Thin light areas of Ni can be seen above the anodic tantalum oxide. It is nickel that has penetrated the pores before their shadowing, as shown in Figure 1.

### 3.3. Crystal Structure Analysis

**Figure 7:**
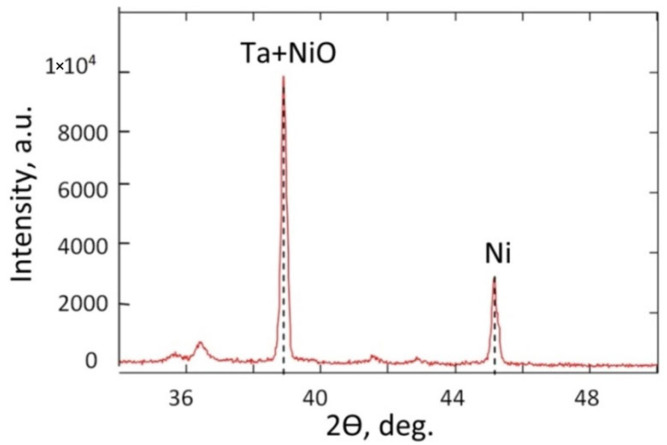
XRD spectrum of the experimental sample of the Ta/Ta_2_O_5_ pillars with Ni tips.

The results of the X-ray structural analysis are provided in Figure 7 and Table 3. According to the JCPDS database (Joint Committee Powder Diffraction Standards, Power Diffraction file, International Centre for Diffraction Data, Pennsylvania, PA, USA, 2001), the main Ta (2θ = 38.37°, PDF-card 25-1280) and Ni (2θ = 44.51°, PDF-card 270-989) peaks were used in the analysis. As the amorphous Ta_2_O_5_ phase, including anodic oxide, transforms into a crystalline form only after annealing at a temperature higher than 800 °C [63,64], it was not detected.

The main Ni peak at 44.51° in the XRD spectra proves the presence of metallic Ni nanoparticles. This diffraction peak corresponds to the (111) Miller index of Ni (cubic structure, space group–Fm3m–PDF-2 card 270-989) according to the JCPDS. Furthermore, the diffraction of the (111) crystal orientation of the NiO phase (PDF-2 card 47-1049) can also contribute to this peak. Nickel oxide can appear because of Ni nanoparticle thermal oxidation during the deposition and vacuum chamber cooling.

### 3.4. Magnetic Properties Study

**Figure 8:**
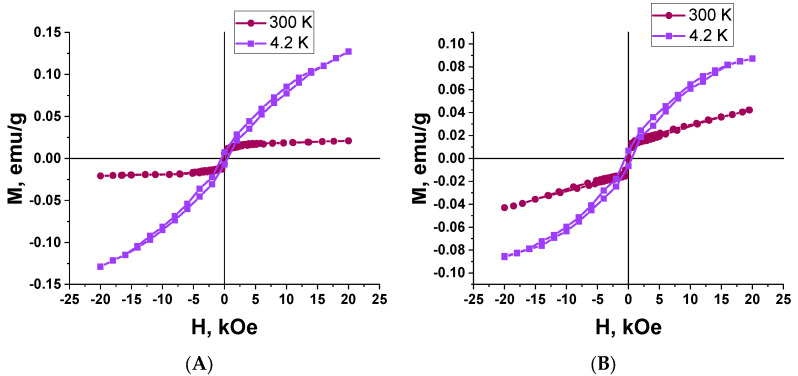
Comparison of the hysteresis loops for two samples of Ta_2_O_5_ pillars with Ni tips at different temperatures: (**A**)—sample 2 (type I), (**B**)—sample 3 (type II).

**Figure 9:**
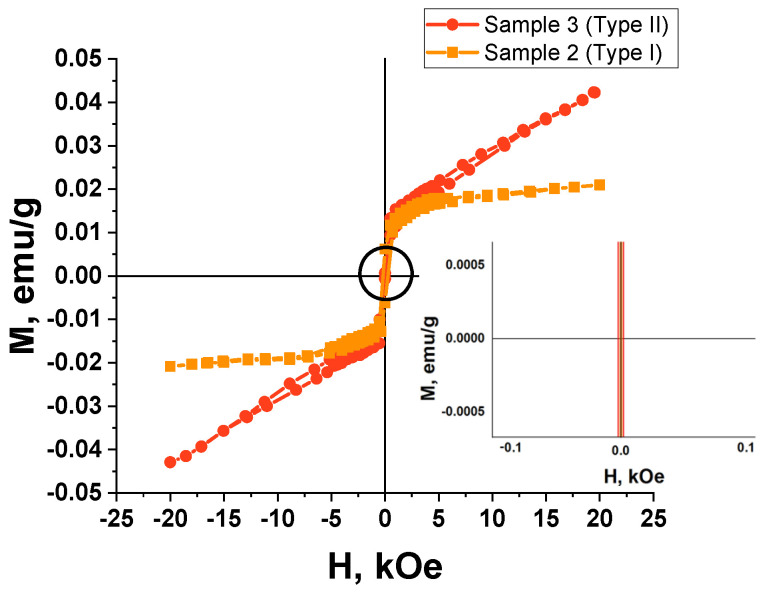
Comparison of the hysteresis loops for two samples of the Ta_2_O_5_ pillars with Ni tips at 300 K.

The results of the magnetic study for two types of the Ta_2_O_5_ pillars with Ni tips are shown in Figure 8 and Figure 9. The samples differ in the diameter and density of the Ta_2_O_5_ pillars. The following samples were investigated: sample 2 with an area of 2.0 × 5.0 = 10 mm^2^, the mass of 0.0181 g, the surface density of 0.181 mg/mm^2^, and the pillar diameter of (40 ± 5) nm; sample 3 with an area of 2.5 × 5.0 = 12.5 mm^2^, the mass of 0.0252 g, the surface density of 0.202 mg/mm^2^, and the pillar diameter of (100 ± 5) nm.

The perpendicular to the surface direction of the magnetic field application (out-of-plane geometry) is parallel to the direction of the Ta_2_O_5_ pillars. As the pillars are diamagnetic and nickel is situated only at their top ends, the field direction is not important, and we chose the parallel field direction.

The dependence of the magnetization on the strength of the magnetic field applied in the sample plane (the in-plane geometry, the parallel to the sample surface direction of the magnetic field application corresponds to the perpendicular to the Ta_2_O_5_ pillar axis direction) was recorded at room temperature (300 K) and 4.2 K with a magnetic field strength sweep up to 20 kOe.

Table 4 includes the calculated main magnetic parameters from the remagnetization curves.

It is important to note that the values of the saturation magnetization M_s_ and the remanent magnetization M_r_ are calculated not per mass of Ni but per the whole mass of the Ta/Ta_2_O_5_/Ni samples, including the mass of the remaining Ta and the mass of the substrate (Si/SiO_2_), as it is difficult to determine the mass and the volume of the metal (Ni) deposited into the TPAA pores. We assume that such a calculation of the specific magnetic properties is a valid approach because all the TPAA substrates prepared for further filling with the pillars were synthesized in the same technological cycle on identical substrates with the same thickness of the Al and Ta layers. The samples studied were taken from the same substrate. Moreover, the signal of a separately measured Si/SiO_2_ substrate was subtracted from the remagnetization curves to consider its contribution.

The absence of magnetic hysteresis (zero coercivity) at room temperature is characteristic for both samples. The absence of hysteresis means that upon switching off the external magnetic field, the particle magnetic moments begin oscillating freely because of thermal fluctuations. It leads to fast nanoparticle demagnetization [65]. It means that the movement of such nanoparticles can be instantaneously controlled with an external magnetic field.

The presented results show that the samples of Ta_2_O_5_ pillars with Ni tips exhibit a weak magnetic response and superparamagnetic properties at room temperature. Superparamagnetism can be seen by the magnetization curve reaching saturation at a comparatively low value of the magnetic field strength (about 1 kOe).

Hence, we can suppose that the magnetic Ni nanoparticles have a super small geometric size of about a few nanometers. In such a case, superparamagnetic properties can be expected for this composite material, as in granulated films [66].

Nickel is a ferromagnetic material. However, the hysteresis loops (Figure 8 and Figure 9) correspond to a superparamagnetic type of magnetism in the samples only. We should note that nickel prepared by different methods, including magnetron deposition, can possess superparamagnetic properties in nanosized dimensions [67]. Small 1 to 10 nm ferro- or ferrimagnetic nanoparticles become single-domain below Curie temperature. In such a state, they behave as permanent magnets, with the magnetization vectors of all their atoms directed equally [68].

We could not determine the thickness of the nickel layer on top of the Ta_2_O_5_ pillars (linear size of the nickel nanoparticles). However, results of the magnetic study suggest that they could have the size at which Ni MNPs exhibit superparamagnetic properties. The effect of ferromagnetism suppression of the nickel MNP can also be related to their oxidation, either during their magnetron deposition onto the substrates at 300 °C or during their investigation at room temperature, as the layer thickness is very low. Hence, we can also suppose that the superparamagnetism is caused by the partial oxidation of nickel crystallites during the deposition or during various room temperature complex studies (that is less probable) because of the oxygen adsorption. Molecular oxygen adsorbed at nickel crystallite boundaries can be the most probable agent leading to the appearance of superparamagnetism in partially oxidized nickel NPs and the composite as a whole, whereas the nanocrystalline nickel itself exhibits ferromagnetism. The size of the nickel nanoparticles is about 15 nm (Table 3). The size of the non-oxidized Ni NPs core can be smaller.

On the one hand, oxidation weakens the ferromagnetic properties of the Ni MNP. On the other hand, it can improve the magnetic order stabilization against thermal fluctuations in comparison with analogous systems of equiaxed magnetic nanoparticles.

The study [11] suggests using magnetic metallic nanoparticles (in particular, iron, cobalt, and nickel) as an alternative to superparamagnetic iron oxide particles in biomedicine. The authors note that besides the materials of MNPs, the most important parameter is the average particle size in combination with their narrow size distribution. To achieve the reproducibility of the properties, the synthesis should be organized so that the formation of single-domain particles with a narrow size distribution is guaranteed. Another important factor is the agglomeration of the particles that should be reduced.

**Figure 10:**
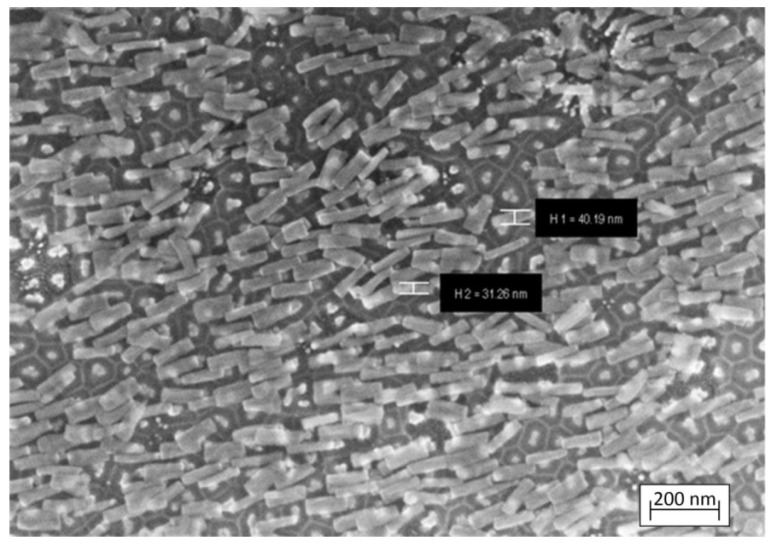
SEM image of the test sample with Ta_2_O_5_/Ni pillars after submersion in a diluted aqueous solution of phosphoric acid (sample 2, type I).

Unlike the long nanowires of pure nickel and cobalt of the same diameter [69,70], the nanopillars from our samples did not group into bundles after submersion in the 4% aqueous H_3_PO_4_ solution (Figure 10). Some pillars had detached from the growth points (Al_2_O_3_ pore bottoms), but all of them had remained on the substrate. 

## 4. Conclusions

We have shown that TPAA templates allow the preparation of aligned nanopillars. By changing the anodizing conditions of the TPAA synthesis, it is possible to change the morphology of the template and, as a result, control the morphology of the nanopillars. We proposed a model of the nanopillar film growth based on field-assisted ionic transport and confirmed it with the experimental data.

The crystal structure analysis showed that the nanopillars mainly consist of amorphous Ta_2_O_5_. The XRD spectra show the presence of metallic nickel nanoparticles (2θ = 44.51). This peak can also include a contribution from the NiO phase with a (111) crystal orientation.

The hysteresis loops of the Ta_2_O_5_ nanopillars with Ni tips are characterized by the absence of magnetic hysteresis at room temperature (zero coercivity). Thus, we can suppose that the nickel nanoparticles have a super small size, 10 nm by order of magnitude. The effect of the ferromagnetism suppression can also be related to oxidation of the nickel nanoparticles either during the magnetron deposition onto the substrates at 300 °C or during the room-temperature investigation, as the layer thickness is very low.

The Ta_2_O_5_ nanopillars with Ni tips separated from the substrate can be easily dispersed in water or ethylene glycol and used in biomedicine. We suggest using the nanostructures prepared in the next-generation nanodevices as energy storage systems [71], chemical and biochemical sensors [72], nanoelectrodes for electrochemical studies in nanosized dimensions [73], etc. Ta_2_O_5_ is biologically inert. Therefore, the Ta_2_O_5_ pillars can be used for medical diagnostics and treatment using short-range quasistationary electric fields, stimulating positive biological properties [74].

Next, we plan to investigate the possibilities of using of fabricated samples for specific applications, for example, in targeted drug delivery systems and in sensor devices (nanodiagnostics, sensitive elements of electrochemical nanosensors). In addition, 2D/3D shapes arrays of nanopillars (nanowires) have wide application possibilities. The combination of this unique structure (the 2D/3D array shapes) with uncommon magnetic, optical and transport properties can be used to develop novel functional nanomaterials for magnetic, electronic, biomedical, and optical nano-scale devices [27,55,75,76,77,78].

## Figures and Tables

**Table 1:** The deposition parameters for the tantalum and aluminum thin films.

Deposition Process Parameters	Tantalum	Aluminum
Residual gas pressure, Pa	1.3 × 10^−3^	1.4 × 10^−4^
Substrate temperature, K	523	423
Accelerating voltage, kV	8	8
Beam current, A	0.4	1.2
Deposition rate, nm·s^−1^	1.0 ± 0.2	5.0 ± 0.5
Thickness, nm	450 ± 50	(1000–2000) ± 50

**Table 2:** Parameters of the TPAA samples and their preparation procedures.

Sample Type	Sample No.	Electrolyte	Voltage, U_Al_, V	Voltage, U_Ta_, V	Pillar (Pore) Diameter, nm	Interpore Distance, nm	Pillar Height, nm	Pillar Density, per cm^2^
type I	1	C_2_H_2_O_4_	40	40	40 ± 5	100 ± 5	150 ± 10	10 × 10^9^
	2			70	40 ± 5	100 ± 5	160 ± 10	10 × 10^9^
type II	3	H_3_PO_4_	80	80	100 ± 5	200 ± 5	180 ± 10	3 × 10^9^
	4			90	100 ± 5	210 ± 5	220 ± 10	2 × 10^9^

**Table 3:** Results of the X-ray structural analysis of the Ta/Ta_2_O_5_/Ni experimental samples. The values in parentheses in the 2θ column are the corresponding angles from the JCPDS database.

Material	Crystallographic Direction	2θ, deg.	I, *%*	Coherence Region Size L, nm
β-Ta	(002)	36.4 (35.61) JCPDS (25-1280)		
α-Ta	(110)	38.37 (38.4) JCPDS (25-1280)	100	-
Ni	(111) face-centered cubic *(fcc)* structure	44.15 (44.51) JCPDS PDF-card (270-989)	32	13
NiO	(111)	37.8 (38.33) JCPDS (47-1049)	-	-

**Table 4:** The results of the magnetic measurements for the samples of the Ta_2_O_5_ pillars with Ni tips.

Type	Sample	Temperature, K	H_c_, kOe	M_r_, emu/g	M_s_, emu/g	M_r_/M_s_
I	2	4.2	511	0.007	0.127	0.06
		300	-	-	-	-
II	3	4.2	525	0.007	0.087	0.08
		300	-	-	-	-

## Data Availability

The data presented in this study are available on request from thecorresponding authors.
