# Correction: Vorobjova et al. A Study of Ta_2_O_5_ Nanopillars with Ni Tips Prepared by Porous Anodic Alumina Through-Mask Anodization. *Nanomaterials* 2022, *12*, 1344

**DOI:** 10.3390/nano15171351

**Published:** 2025-09-02

**Authors:** Alla I. Vorobjova, Daria I. Tishkevich, Elena A. Outkina, Dmitry L. Shimanovich, Ihar U. Razanau, Tatiana I. Zubar, Anastasia A. Bondaruk, Ekaterina K. Zheleznova, Mengge Dong, Dalal A. Aloraini, M. I. Sayyed, Aljawhara H. Almuqrin, Maxim V. Silibin, Sergey V. Trukhanov, Alex V. Trukhanov

**Affiliations:** 1 Micro- and Nanoelectronics Department, Belarusian State University of Informatics and Radioelectronics, 220013 Minsk, Belarus; vorobjova@bsuir.by (A.I.V.); outkina@bsuir.by (E.A.O.); shdl@tut.by (D.L.S.); katenickerd@gmail.com (E.K.Z.); 2 Scientific-Practical Materials Research Centre of NAS of Belarus, 220072 Minsk, Belarus; ir23.by@gmail.com (I.U.R.); fix.tatyana@gmail.com (T.I.Z.); bondaruk625@gmail.com (A.A.B.); sv_truhanov@mail.ru (S.V.T.); truhanov86@mail.ru (A.V.T.); 3 Laboratory of Single Crystal Growth, South Ural State University, 454080 Chelyabinsk, Russia; 4 Department of Resource and Environment, Northeastern University, Shenyang 110819, China; mg_dong@163.com; 5 Department of Physics, College of Science, Princess Nourah Bint Abdulrahman University, P.O. Box 84428, Riyadh 11671, Saudi Arabia; daalorainy@pnu.edu.sa (D.A.A.); ahalmoqren@pnu.edu.sa (A.H.A.); 6 Department of Physics, Faculty of Science, Isra University, Amman 1162, Jordan; dr.mabualssayed@gmail.com; 7 Department of Nuclear Medicine Research, Institute for Research and Medical Consultations (IRMC), Imam Abdurrahman bin Faisal University (IAU), Dammam 31441, Saudi Arabia; 8 Scientific and Technological Park of Biomedecine, I.M. Sechenov First Moscow State Medical University, 119991 Moscow, Russia; sil_m@mail.ru

In the original publication [1], reference [4], cited in the first sentence of Section 1. Introduction, was incorrectly attributed to (“Kozlovskiy, A.L.; Kenzhina, I.E.; Zdorovets, M.V.; Saiymova, M.; Tishkevich, D.I.; Trukhanov, S.V.; Trukhanov, A.V. Synthesis, phase composition and structural and conductive properties of ferroelectric microparticles based on ATiOx (A = Ba, Ca, Sr)” [2]).

The citation [4] has now been corrected in the reference list as follows [3]:

“Tsurin, V.A.; Yermakov, A.Y.; Uimin, M.A.; Mysik, A.A.; Shchegoleva, N.N.; Gaviko, V.S.; Maikov, V.V. Synthesis, structure, and magnetic properties of iron and nickel nanoparticles encapsulated into carbon. *Phys. Solid State* **2014**, *56*, 287–301. doi:10.1134/S1063783414020309.”

With this correction, the order of references remains unchanged. The authors state that the scientific conclusions are unaffected. All authors have read and agreed to the published version of the manuscript. This correction was approved by the Academic Editor. The original publication has also been updated.
